# Inclusion of imputed genotypes from non-genotyped dairy cattle in a Thai multibreed genomic-polygenic evaluation

**DOI:** 10.5713/ab.24.0317

**Published:** 2024-10-24

**Authors:** Danai Jattawa, Thanathip Suwanasopee, Mauricio A. Elzo, Skorn Koonawootrittriron

**Affiliations:** 1 Department of Animal Science, Faculty of Agriculture, Kasetsart University, Bangkok 10900, Thailand; 2 Tropical Animal Genetic Special Research Unit (TAGU), Kasetsart University, Bangkok 10900, Thailand; 3 Department of Animal Sciences, University of Florida, FL 32611, USA

**Keywords:** Accuracy, Genome, Imputation, Prediction, Tropics

## Abstract

**Objective:**

This study assessed the impact of incorporating imputed single nucleotide polymorphism (SNP) information from non-genotyped animals on genomic-polygenic evaluations in a Thai multibreed dairy population under various levels of imputation accuracy.

**Methods:**

Data encompassed pedigree and phenotypic records for 305-day milk yield (MY), 305-day fat (Fat), and age at first calving (AFC) from 12,859 first-lactation cows, and genotypic records of various densities from 4,364 animals. A set of 64 animals genotyped with GeneSeek Genomic Profiler 80K and with four or more genotyped progenies was defined as target animals to simulate imputation scenarios for non-genotyped individuals. Actual and imputed genotypes were utilized to construct three SNP sets. All SNP Sets contained actual and imputed SNP markers from genotyped animals. SNP Set 1 contained no SNPs from target animals, whereas SNP Set 2 incorporated imputed SNPs from target animals, and SNP Set 3 added actual SNPs from target animals. Genomic-polygenic evaluations were conducted using a 3-trait single-step model that included contemporary group, calving age, and heterozygosity as fixed effects and animal additive genetic and residual as random effects.

**Results:**

The imputation accuracy was similar across non-genotyped animals irrespective of the number of genotyped progenies (average: 40.55%; range: 34.68% to 53.82%). Estimates of additive genetic and environmental variances and covariances for MY and AFC varied across SNP sets. SNP Sets 1 and 2 had slightly higher additive genetic and lower environmental variances and covariances than SNP Set 3. Heritabilities and additive genetic, environmental, and phenotypic correlations between MY, Fat, and AFC were similar across all SNP Sets. Spearman rank correlations between genomic-polygenic estimated breeding values from SNP Sets 2 and 3 were high for all traits (0.9990±0.0003).

**Conclusion:**

Utilization of phenotypic and pedigree data from imputed non-genotyped animals enhanced the efficiency and cost-effectiveness of the genetic improvement program in the Thai multibreed dairy cattle population.

## INTRODUCTION

Genomic information has played an increasingly important role in the characterization and evaluation of dairy cattle. The acquisition of genotypic data, often comprising single nucleotide polymorphisms (SNPs), has engendered the development of diverse genotyping chips designed to facilitate accessibility to such information by researchers, farmers, and stakeholders. These genotyping chips, offered in various SNP densities, addressed concerns regarding genotyping costs, with lower-density (LD) options particularly instrumental. However, studies on dairy genomic evaluations consistently demonstrated the superiority of high-density (HD) chips in accurately evaluating economically important traits compared to their LD counterparts [1–4]. To mitigate costs while maintaining evaluation accuracy, a promising strategy involved initially genotyping animals with LD chips and imputing their genotypes to HD chips before genomic evaluation [2]. This practical implementation typically involved genotyping a reference population comprising key ancestors with HD chips, while animals from subsequent generations were genotyped using LD chips, thus achieving a cost-effective level of accuracy in genomic evaluations.

Several recent investigations have incorporated imputation techniques into dairy genome-wide association and genomic evaluation studies. These studies indicated that the accuracy of genomic imputation from LD to HD chips in purebred dairy populations ranged from 81% to 99% [2,5,6]. This variability was contingent upon the specific imputation methods employed and the genetic structure of the cattle population. Notably, Jattawa et al [7] documented genomic imputation accuracies ranging from 77% to 94% when imputing from LD to moderate-density chips in the Thai multibreed dairy population. Furthermore, the combination of actual and imputed genotypes yielded prediction accuracies comparable to those achieved using only actual genomic information in the Thai dairy cattle population [8].

Imputing genomic information from LD to HD chips enhances genomic prediction accuracy and cost-effectiveness in a dairy cattle population and can also be used to predict genomic information for non-genotyped animals. This relies on non-genotyped animals exhibiting sufficient genetic relatedness to genotyped individuals. Multiple studies have demonstrated the feasibility of imputing SNP data for non-genotyped dairy cattle, both through simulated data (accuracy ranging from 0.78 to 0.99; [9–11] and actual data (accuracy ranging from 0.47 to 0.99; [2,9]). However, no research exists in Thailand on the impact of SNP imputation accuracy on multibreed dairy genomic evaluations that utilize imputed non-genotyped animals. In addition, 172 out of 5,003 animals (3.34%) in the current Thai dairy dataset used for genomic-polygenic evaluation lack genotypic information, but their genotypes can be imputed [12]. Using these imputed genotypes could significantly enhance the efficiency of the Thai breeding program. Thus, this study aimed to assess the impact of incorporating imputed SNP information from non-genotyped animals on genomic-polygenic evaluations in a Thai multibreed dairy population under various levels of imputation accuracy.

## MATERIALS AND METHODS

### Animals, data, and traits

The dataset utilized in this research was derived from actual routine genomic-polygenic evaluations in the Thai multibreed dairy population. The dataset included 12,859 first-lactation cows with a pedigree comprising 23,254 animals (1,599 sires and 21,655 cows). These cows calved in 1,190 farms across five Thai regions (North, Northeastern, Western, Central, and Southern) between 1989 and 2019. Thailand exhibits a tropical climate shaped by two predominant monsoons. The southwest monsoon occurs from May to September, bringing about extensive rainfall nationwide.

In contrast, the northeast monsoon happens between October and February, contributing to cold and arid conditions in most regions. The combined influence of these monsoons gives rise to generally elevated temperatures and humidity levels. The daily average ambient temperature typically ranges from 26°C to 29°C, with relative humidities between 73% and 76%. However, regional and seasonal variations exist. In the summer (March to June), average temperatures range from 28.1°C to 29.7°C, and humidity varies between 63% and 75%. During the rainy season (July to October), average temperatures fluctuate between 27.3°C and 28.3°C, with relative humidity levels ranging from 78% to 81%. In winter (November to February), average temperatures range from 23.4°C to 26.7°C, with relative humidity levels fluctuating between 69% and 74% [13].

The prevailing tropical climate conditions and the inherent challenges associated with smallholder farming management have led to adoption of a strategic upgrading approach. Local cattle breeds (*Bos indicus*), including Thai native, Red Sindhi, Sahiwal, and Brahman were impregnated with semen from straightbred and crossbred Holstein, as well as other *Bos taurus* including Jersey, Brown Swiss, and Red Dane [14]. This strategic shift was implemented to improve productivity while ensuring adaptability to tropical environmental conditions. Consequently, the Thai dairy population evolved into a multibreed dairy population, where animals can have genetic material coming from one to eight breeds, although their average genetic makeup is from only three breeds [15]. The dominant breed in the Thai dairy population is Holstein, followed by Brown Swiss, Jersey, Red Dane, Thai native, Sahiwal, Red Sindhi, and Brahman. The Holstein fraction of cows accounts for an average of 87.98% (standard deviation [SD] = 10.92%). Approximately 93% of cows, 96% of sires, and 88% of dams have a genetic makeup of 75% Holstein or higher.

The traits investigated in this research were 305-day milk yield (MY; kg), 305-day fat (Fat; %), and age at first calving (AFC; month). The MY and Fat were recorded in individual cows monthly, from the fifth day post-calving to the end of lactation. These test-day milk records were used to compute MY and Fat. The computation of MY involved the application of the test interval method [16,17]. Conversely, Fat was computed using average test-day fat measurements from the first to the eleventh record at approximately the 305th days in milk.

### DNA samples and genotypes

The animal use protocol for this research was approved by the Institutional Animal Care and Use Committee of Kasetsart University, Thailand (ACKU64-AGR-025). Tissue samples, including blood and semen, were collected from 4,364 animals (143 sires, 434 dams, and 3,787 cows) across 475 farms.

**Figure 1:**
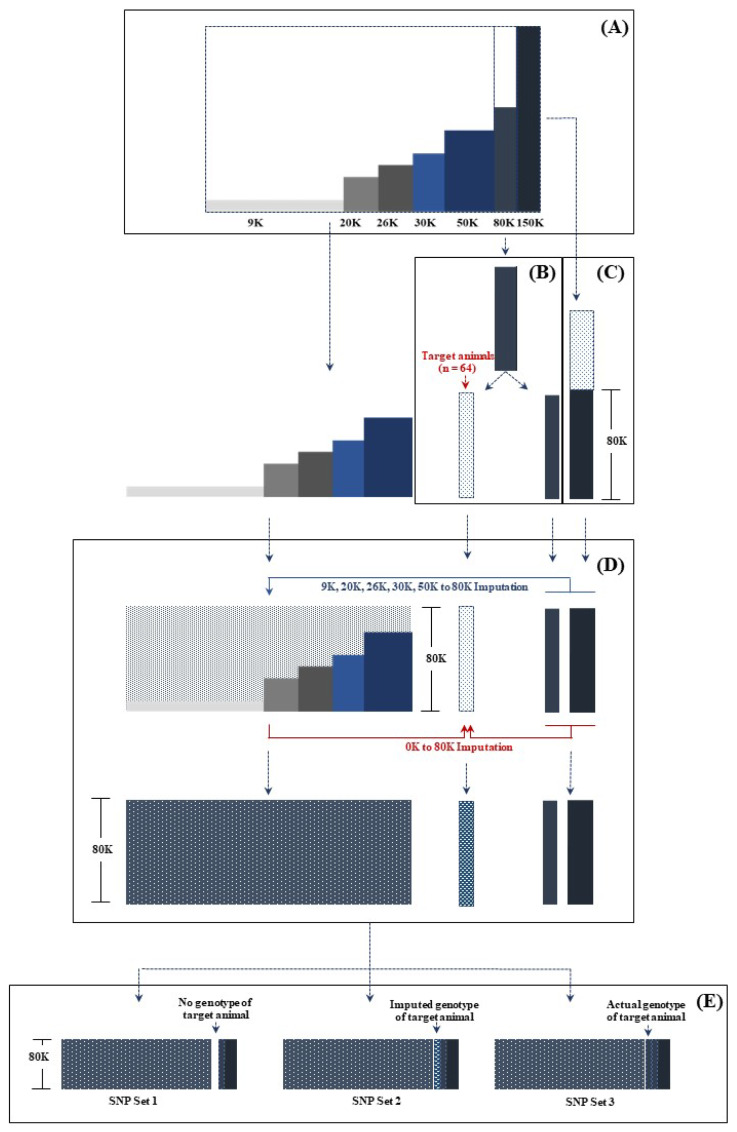
Genotypic information, imputation scenarios, and genotypic datasets used in this research. (A) Dairy cattle (n = 4,364) were genotyped with GeneSeek Genomic Profiler 9K (n = 1,412), 20K (n = 570), 26K (n = 540), 30K (n = 563), 50K (n = 887), 80K (n = 139), and 150K (n = 253). (B) Target animals (n = 64) were selected from 80K genotyped animals, and assumed to be ungenotyped animals by eliminating their SNP genotypes. (C) Animals genotyped with 150K were assumed to have been genotyped only for the subset of SNP contained in the 80K chip. (D) Genotypic imputation was carried out from five low (9K) and moderate (20K, 26K, 30K, and 50K) density chips to 80K. Subsequently, the actual and imputed 80K SNP were used to impute 80K unknown genotypes of target animals. (E) The actual and imputed 80K SNP datasets (n = 70,795 SNPs) from 4,364 animals were used to construct three SNP sets: SNP Set 1, actual and imputed SNP markers from genotyped animals, and no SNP markers from target animals; SNP Set 2, actual and imputed SNP markers from genotyped animals plus imputed SNP markers from target animals; and SNP Set 3, actual and imputed SNP markers from genotyped animals plus actual SNP markers from target animals. SNP, single nucleotide polymorphism.

The average number of progeny with tissue samples was 16.83 for sires (0 to 158) and 1.24 for dams (1 to 3). The DNA was extracted from tissue samples using the MasterPure DNA Purification Kit (Epicentre, Madison, WI, USA). DNA quality and concentration were evaluated using a NanoDrop 2000 spectrophotometer (Thermo Scientific, Wilmington, DE, USA). Only DNA samples exhibiting a concentration surpassing 15 ng/μL and an absorbance ratio of approximately 1.8 at 260/280 nm were deemed acceptable and subsequently forwarded to GeneSeek (GeneSeek Inc., Lincoln, NE, USA) for genotyping. All DNA samples from sires (n = 143) and highly related cows (n = 249) were genotyped with HD GeneSeek Genomic Profiler chips ([GGP]150K: n = 253; GGP80K: n = 139). The remaining cows (n = 3,972) were genotyped with moderate-density (GGP50K: n = 887; GGP30K: n = 563), and LD (GGP26K: n = 540; GGP20K: n = 570: GGP9K: n = 1,412) GeneSeek GGP (Figure 1A). The numbers of SNP genotypes in autosomes and the X chromosome were 8,590 for the GGP9K, 19,616 for GGP20K, 25,979 for GGP26K, 29,795 for GGP30K, 44,955 for GGP50K, 76,694 for GGP80K, and 124,926 for GGP150K.

### Genotypic imputation

To assess the imputation accuracy for non-genotyped animals, it is essential to have access to the actual genotypes of the target animals slated for imputation. Additionally, the target animals should ideally possess four or more genotyped progenies in accordance with the guidelines specified in the default settings for imputation programs such as Findhap [18] and FImpute [5]. However, the genotyped animals employed in this research were drawn from real scenarios, reflecting routine genomic evaluations conducted within the Thai dairy population. Genotyping chips of seven densities were used to acquire genomic information from animals in this population (Figure 1A). Assignment of chips to animals was contingent on: 1) relationships among genotyped animals and other animals in the population; highly related animals were genotyped with a HD chip, and LD chips were used in the remaining animals; 2) availability of chips during specific genotyping periods (e.g., 2013: HD = GGP80K, LD = GGP9K; 2015: HD = 80K, LD = GGP20K and GGP26K; 2019: HD = GGP150K, LD = 50K; 2021: HD = GGP150K, LD = GGP100K); and 3) genotyping budget allocated for a particular year. Unfortunately, a significant proportion of highly related animals suitable for designation as target animals for non-genotype imputation were genotyped with a GGP80K chip. This limitation determined the imputation from 9K, 20K, 26K, 30K 50K to 80K to assess imputation accuracy for non-genotyped animals in this study.

**Figure 2:**
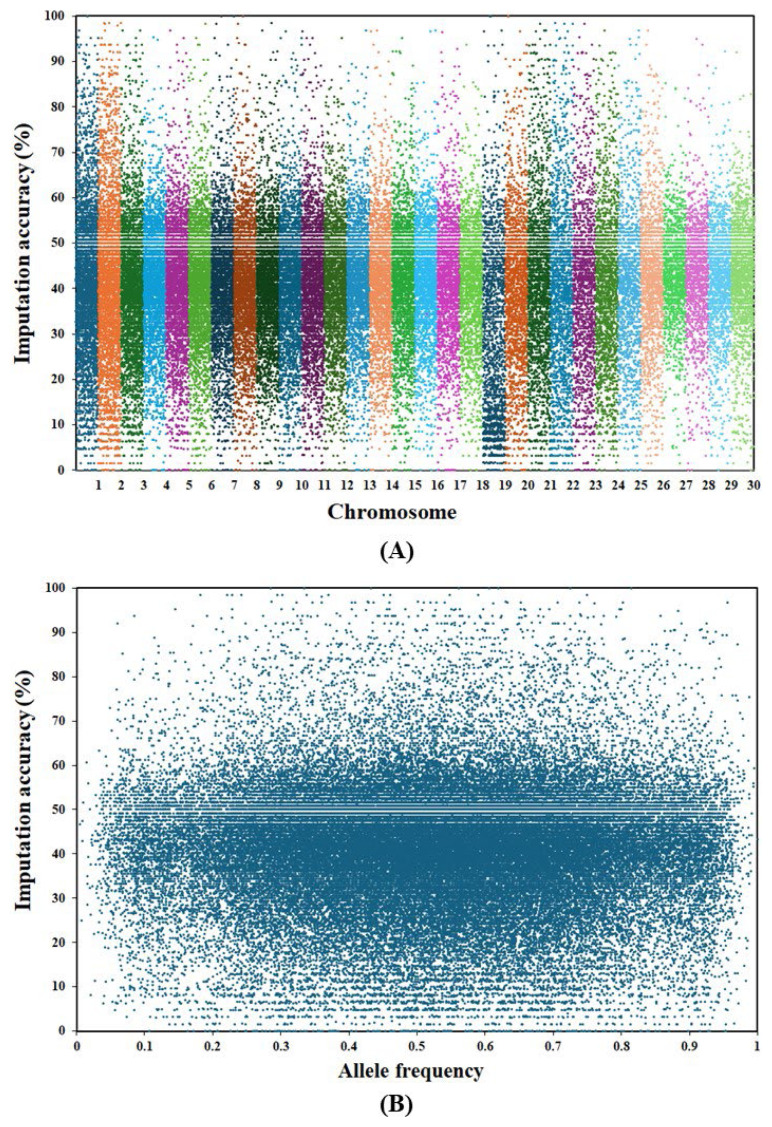
Imputation accuracy for non-genotyped animals in the Thai multibreed dairy cattle population classified by (A) SNP markers within chromosome and (B) allele frequency. SNP, single nucleotide polymorphism.

The genomic preparation and imputation process were executed in the following manner: 1) animals genotyped with GGP80K, possessing four or more genotyped progenies, were designated as target animals (n = 64), and treated as ungenotyped by eliminating their SNP genotypes. The remaining GGP80K genotyped animals (n = 75) were used as the reference population, along with the genotyped animals managed in the next step (Figure 1B); 2) animals genotyped with GGP150K were assumed to be genotyped with GGP80K by selecting only the subset of SNPs contained in GGP80K (Figure 1C); and 3) genotypic imputation was conducted from the LD (9K) and moderate-density (20K, 26K, 30K, and 50K) chips to the 80K chip. Subsequently, the actual and imputed 80K genotypes were employed to impute the unknown genotypes of target animals (Figure 1D). The genomic imputation utilized combined family- and population-based algorithms from Findhap 4.0 [18]. The choice of Findhap 4.0 for this research was influenced by several factors: 1) its suitability and capacity to deliver high imputation accuracy (exceeding 80%) for the Thai dairy population [7], 2) its capability to impute genotypes for ungenotyped animals, and 3) its open accessibility and usage without cost ( https://aipl.arsusda.gov/software/findhap).

After the imputation process was completed, imputed genotypes from target animals were compared to their actual genotypes to ascertain the accuracy of non-genotyped imputation. This assessment involved computing the ratio of correctly imputed SNP markers to the total number of imputed SNP markers across the 64 target animals. Missing SNP markers in all animals were excluded from accuracy computations.

### Genomic-polygenic evaluations

Actual and imputed genotypes were utilized to construct three sets of SNP markers to investigate the impact of imputation accuracy in non-genotyped target animals on genomic-polygenic evaluation (Figure 1E). These three SNP sets included: 1) actual and imputed SNP markers from genotyped animals only, and no SNP markers from target animals (SNP Set 1); 2) actual and imputed SNP markers from genotyped animals plus imputed SNP markers from target animals (SNP Set 2); and 3) actual and imputed SNP markers from genotyped animals plus actual SNP markers from target animals (SNP Set 3). Thus, these three SNP sets differed only in the SNP markers from the target animals. SNP markers with minor allele frequencies lower than 0.04 or call rates lower than 0.9 were eliminated from the three SNP sets (n = 70,795 SNPs). Consequently, the numbers of retained SNP were 68,377 SNPs (4,300 animals) in SNP Set 1, 70,240 (4,364 animals) in SNP Set 2, and 70,307 (4,364 animals) in SNP Set 3.

Each set of SNPs was utilized with pedigree and phenotypes to estimate variance-covariance components and genetic parameters as well as to compute genomic-polygenic estimated breeding values (GPEBV) for MY, Fat, and AFC. Estimates of variance-covariance components were obtained using a 3-trait single-step genomic-polygenic model [19]. Fixed effects for this model included contemporary group (herd-year-season) and heterosis (as a linear function of Holstein-Other breeds heterozygosity). Heterozygosity was computed as the product of Holstein fraction in the sire multiplied by Other breeds fraction in the dam plus Other breeds fraction in the sire multiplied by Holstein fraction in the dam. Random effects were animal additive genetic and residual. The mean for random effects was assumed to be zero. The variance-covariance matrix among animal additive genetic effects for MY, Fat, and AFC was equal to H⊗V_a_, where H was the genomic-polygenic relationship matrix, V_a_ was a 3×3 matrix of additive genomic-polygenic variances and covariances among traits, and ⊗ was the Kronecker product. Matrix H was equal to:


$$\left[\begin{array}{cc} {\text{A}}_{11}+{\text{A}}_{12}{\text{A}}_{22}^{-1}\left({\text{G}}_{22}-{\text{A}}_{22}\right){\text{A}}_{22}^{-1}{\text{G}}_{21} & {\text{A}}_{12}{\text{A}}_{22}^{-1}{\text{G}}_{22}\\ {\text{G}}_{22}{\text{A}}_{22}^{-1}{\text{A}}_{21} & {\text{G}}_{22} \end{array}\right],$$

where A_11_ = submatrix of additive relationships among non-genotyped animals, A_12_ = submatrix of additive relationships among non-genotyped and genotyped animals, ${\text{A}}_{22}^{-1}$ = inverse of the additive relationship submatrix for genotyped animals, G_22_ = matrix of genomic relationships among genotyped animals [20]. Matrix G_22_ was computed as ZZ′/2∑p_j_(1−p_j_), where p_j_ = frequency of allele 2 in locus j, *z_ij_* = (0−2*p_j_*) for genotype = 11 in locus j, *z_ij_* = (1−2*p_j_*) for genotype = 12 and 21 in locus j, and *z_ij_* = (2−2*p_j_*) for genotype = 22 in locus j [19,21]. Matrix G_22_ was scaled based on matrix A_22_ using the default parameters of the BLUPF90 Family of Programs [22], i.e., mean of the diagonal elements of G_22_ = mean of the diagonal elements of A_22_, and mean of off-diagonal elements of G_22_ = mean of off-diagonal elements of A_22_. The variance-covariance matrix among residuals was equal to I⊗V_e_, where I was the identity matrix, and V_e_ was a 3×3 matrix of residual variances and covariances among traits.

Variances for and covariances between MY, Fat, and AFC were estimated using an average information restricted maximum likelihood algorithm with program AIREMLF90 [23] of the BLUPF90 Family of programs. Standard errors for additive genetic and environmental variances and covariances were computed as square roots of their estimated error variances obtained from the diagonal elements of the inverse of the average information matrix. The repeated sampling procedure [24] option within AIREMLF90 was used to estimate phenotypic variances and covariances, heritabilities, and their standard deviations. In addition, phenotypic, genetic, and environmental correlations between MY, Fat, and AFC and their standard deviations were also estimated using repeated sampling procedures.

Animal GPEBV for MY, Fat, and AFC were predicted using single-step genomic BLUP (ssGBLUP; [19]) with program BLUPF90 [22]. The estimates of variances, covariances, and H matrices for the three SNP sets (Figure 1E) were used for ssGBLUP. All animal rankings based on their GPEBV for MY, Fat, and AFC from the three SNP sets were compared using Spearman’s rank correlations computed using the CORR procedure of SAS OnDemand for Academics version (SAS Institute Inc., Cary, NC, USA).

## RESULTS AND DISCUSSION

### Imputation accuracy for non-genotyped animals

The accuracy of imputation for non-genotyped animals using various reference chips in the Thai multibreed dairy cattle population is presented in Table 1. On the average, there were 1,728,235 correctly imputed SNP out of a total of 4,261,532 imputed genomic SNP in the 64 non-genotyped animals, resulting in an average imputation accuracy of 40.55%. Imputation accuracies ranged from 34.68% to 53.82%. These imputation accuracies were relatively modest compared to those reported in straightbred Holstein dairy cattle (47% to 99%; [2,9]) and Nelore beef cattle populations (81.6% to 97.4%; [9]). The lower accuracies obtained here may be attributed to several factors. Firstly, differences in population structure may have played a crucial role. The Thai multibreed population, consisting of Holstein and seven other *Bos taurus* and *Bos indicus breeds*, exhibited lower linkage disequilibrium than purebred populations [25]. The average linkage disequilibrium between adjacent SNP markers (r^2^) across the 30 autosomes was 0.036, with values ranging from 0.000 to 0.982. The lower linkage disequilibrium observed in the Thai multibreed population signifies shorter shared haplotype lengths. Perhaps this is impairing the efficiency and accuracy of haplotype phasing and subsequent imputation processes [5,26].

Imputation accuracies for non-genotyped animals were assessed across all SNPs on autosomes and across all allele frequencies (Figure 2). The analysis revealed that these accuracies were distributed without a discernible pattern, both across chromosomes (Figure 2A) and across allele frequencies (Figure 2B). This indicated that SNPs and allele frequencies had only a minor influence on imputation accuracy for non-genotyped individuals in this population. Instead, factors such as degree of relatedness among individuals and number of genotyped progenies may have had a more substantial impact. The absence of a clear pattern in imputation accuracy highlighted the complexity of factors affecting imputation performance and underscored the need for further investigation to identify and understand these determinants.

This study employed the combined family- and population-based approach from the Findhap program routinely used in the genomic evaluation of the Thai multibreed dairy population. This approach is known for its high accuracy when imputing from low to moderate density chips in Holstein-upgraded dairy cattle in Thailand [7]. Compatibility between the imputation method and population structure was found to be essential for improving imputation accuracy. VanRaden et al [2] showed that FImpute provided higher accuracy than Findhap for dairy cattle populations in the US, Canada, Britain, and Italy. Further, Bouwman et al [10] reported that AlphaImpute surpassed Findhap in the Dutch dairy population. These findings emphasize the need to identify suitable imputation methods for the Thai dairy population to enhance the accuracy of genotype imputation for non-genotyped animals and to increase the effectiveness of future dairy breeding programs.

**Figure 3:**
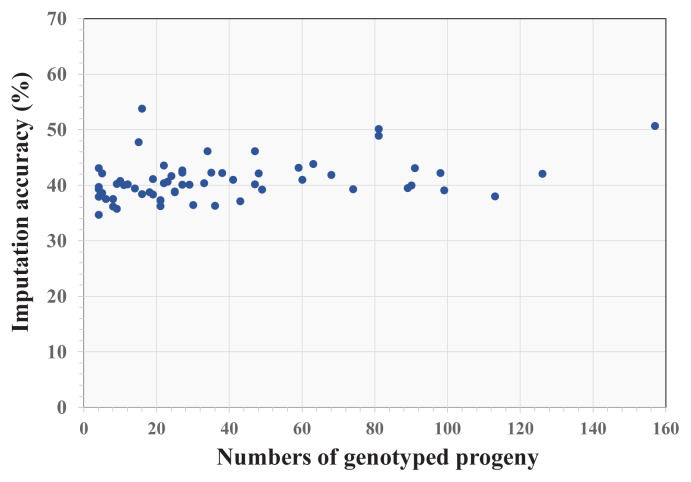
Imputation accuracy for non-genotyped animals in the Thai multibreed dairy cattle population classified by number of genotyped progeny.

The degree of kinship between genotyped and non-genotyped animals is another pivotal factor that may influence the genomic SNP imputation accuracy in non-genotyped animals. Prior research showed that including a larger number of genotyped progenies from non-genotyped animals would improve imputation accuracy [10,27,28]. The average relationship between animals in the target and reference populations was 0.0005 (SD = 0.009), with minimum and maximum values of 0.000 and 0.625, respectively. However, imputation accuracies in the 64 non-genotyped animals from the Thai multibreed population tended to be fairly similar irrespective of their number of genotyped progeny (Figure 3). The animal with the highest imputation accuracy (53.82%) was associated with 16 genotyped progenies, whereas the animal with the lowest imputation accuracy (34.68%) had only four genotyped progenies. Conversely, the animal with the largest cohort of 4 indicate that numbers of genotyped progeny in the Thai multibreed population may have less influence on imputation accuracy than in other cattle populations.

This outcome prompted a deeper examination of the intricacies influencing SNP imputation accuracy in non-genotyped animals. The lack of a consistent positive association between a number of genotyped progeny and imputation accuracy in the Thai multibreed population suggests a more complex relationship between non-genotyped animals and their progeny regarding imputation accuracy and perhaps the involvement of additional influencing factors. Genetic diversity within contributing breeds [29,30], quality of genotypic data [31], number of animals in the reference population [5], and imputation algorithms [2,10] may have played some role in the imputation accuracies obtained in the Thai dairy population. Thus, an approach that accounts for genetic and non-genetic factors may provide a clearer justification for imputation accuracy values obtained in multibreed cattle populations.

### Variance-covariance components, heritabilities, and genetic correlations

Estimates of variances and covariances for MY, Fat, and AFC from genomic-polygenic evaluations using three sets of SNP markers are shown in Table 2 for additive genetic effects, Table 3 for environmental effects, and Table 4 for phenotypic effects. Estimates of phenotypic variances and covariances for MY, Fat, and AFC were comparable across the three SNP sets. In addition, similar estimates of additive genetic and environmental variances for Fat, as well as their covariances with other traits, were obtained for all SNP sets. However, estimates of additive genetic variances and covariances for MY and AFC from SNP Set 1 (MY: 179,150 kg^2^; AFC: 4.90 mo^2^; covariance: −1.27 kg*mo) and SNP Set 2 (MY: 170,160 kg^2^; AFC: 4.81 mo^2^; covariance: −3.00 kg*mo) were slightly higher than those obtained with SNP Set 3 (MY: 155,710 kg^2^; AFC: 4.44 mo^2^; covariance: −0.12 kg*mo). Conversely, estimates of environmental variances for MY from SNP Set 1 (413,410 kg^2^) and SNP Set 2 (419,950 kg^2^) were somewhat lower than the corresponding value from SNP Set 3 (431,860 kg^2^). Lastly, similar estimates of environmental variances (16.68 mo^2^ to 17.02 mo^2^) and covariances between MY and AFC (169.21 kg*mo to 173.18 kg*mo) existed across the three SNP Sets. Thus, inclusion of imputed genotypes from non-genotyped animals resulted in slightly higher estimated of additive genetic variances and covariances and slightly lower estimated of environmental variances and covariances.

Heritabilities and additive genetic, environmental, and phenotypic correlations between MY, Fat, and AFC are shown in Table 5. Moderate heritabilities were estimated for all traits in all SNP Sets. Heritability estimates for MY ranged from 0.27±0.04 (SNP Set 3) to 0.30±0.04 (SNP Set 1). Heritability estimates for Fat were identical across all SNP sets (0.27±0.04). Heritability estimates for AFC ranged from 0.21±0.04 (SNP Set 3) to 0.23±0.04 (SNP Set 1). In addition, similar estimates of additive genetic, environmental, and phenotypic correlations between MY, Fat, and AFC were obtained in all SNP Sets. Despite the variability among estimates of additive genetic variances and covariances, heritability estimates for MY, Fat, and AFC exhibited remarkable consistency across all SNP sets, with moderate values highlighting the balanced contribution of genetic and environmental factors to these traits. This consistency indicated that the fundamental genetic architecture of these traits was effectively captured regardless of the specific SNP marker composition. Further, the similarity among estimates of heritabilities and additive genetic correlations between MY, Fat, and AFC across SNP sets reinforced the notion that, although important, the selection of SNP markers did not drastically affect the estimation of genetic parameters for these traits in the Thai multibreed dairy population.

### Animal rankings

Spearman rank correlations among genomic-polygenic estimated breeding values (EBVs) for MY, Fat, and AFC predicted using SNP Sets 1, 2, and 3 are reported in Table 6. The highest rank correlations were observed between genomic-polygenic EBVs predicted from SNP Sets 2 and 3 (0.9990±0.0003 for MY, Fat, and for AFC). The second-highest rank correlations were between genomic-polygenic EBVs predicted from SNP Sets 1 and 3 (0.9769±0.0014 for MY, 0.9480±0.0021 for Fat, and 0.9518±0.0020 for AFC). The lowest rank correlations were those between genomic-polygenic EBVs obtained from SNP Sets 1 and 2 (0.9759±0.0014 for MY and 0.9470±0.0021 for Fat and 0.9528±0.0020 for AFC).

Rank correlations between SNP Sets exceeded 0.9000 (p<0.0001) for all traits, indicating a remarkable level of consistency among genomic-polygenic EBVs from the three SNP Sets. The variation among rank correlations could be partly attributed to differences among genomic-polygenic EBV from the three SNP Sets due to differences in estimated additive genetic (Table 2) and environmental (Table 3) variances and covariances. In addition, the effect of the three SNP Sets on the values of the elements of the genomic-polygenic relationship matrix H likely contributed to differences among animal genomic-polygenic EBV across SNP Sets.

The high rank correlations (0.9990) between animal genomic-polygenic EBVs from SNP Sets 2 and 3 pointed out the benefit of including phenotypic data from non-genotyped animals in the computation of genomic-polygenic EBVs. These outcomes underscored the importance of maintaining accurate dairy datasets, highlighting their ability to enhance breeding program efficacy and cost-effectiveness in Thailand.

This study corroborated previous findings indicating that integration of phenotypes from individuals with imputed genotypes into the reference population of genotyped individuals could enhance the accuracy of genomic predictions [32–34]. Pimentel et al [34] emphasized that the extent of the improvement in prediction accuracy tended to be more pronounced for traits with lower heritabilities and in cases where the initial reference population was limited in size. However, the effectiveness of imputation for bolstering prediction accuracy was contingent upon several factors, including the precision of the imputation process and the structure of the population under study. Pszczola et al [35] found that a low imputation accuracy could impede significant enhancements in prediction accuracy, particularly in populations characterized by complex structures where only specific individuals, such as sires and maternal grandsires, possessed genotypic information. Similarly, Hickey et al [33] suggested that imputing genotypes for ungenotyped individuals with distant relationships to the genotyped population may not substantially improve prediction accuracy.

Results from this study indicate that incorporating imputed genotypes from non-genotyped animals into genomic-polygenic evaluations would be advantageous to increase the accuracy of genomic-polygenic predictions in the Thai dairy multibreed population. The high Spearman rank correlations between GEBVs from SNP Sets 2 and 3 further underscored the potential utility of incorporating phenotypic and pedigree data from non-genotyped animals for predicting animal genomic-polygenic EBVs with accuracy comparable to that achieved by using data from genotyped animals. This finding highlighted the promising nature of this strategy to enhance the efficacy and cost-effectiveness of dairy genetic improvement programs in Thailand.

In conclusion, including imputed genotypes from non-genotyped animals in genomic-polygenic evaluations represents a pivotal step towards enhancing the accuracy of genetic predictions in the Thai multibreed dairy population. The imputation of non-genotyped animals using various reference chips in the Thai multibreed dairy population was feasible, with an average imputation accuracy of 40.55% (ranging from 34.68% to 53.82%). The imputation accuracies obtained remained consistent across all non-genotyped animals, regardless of their number of genotyped progeny. Integrating imputed genotypes from non-genotyped animals into genomic-polygenic evaluations for MY, Fat, and AFC yielded estimates of variance components and genetic parameters similar to those obtained using actual SNPs. The high Spearman rank correlations between genomic-polygenic EBVs from SNP Sets 2 and 3 emphasized the advantage of using phenotypic and pedigree data from non-genotyped animals to increase the effectiveness of the genetic improvement program in the Thai multibreed dairy cattle population.

## Figures and Tables

**Table 1:** Accuracy of imputation for non-genotyped animals in the Thai multibreed dairy cattle population

Number of non-genotyped imputed animals	64
Average total number of imputed SNP	4,261,532
Average number of correctly imputed SNP	1,728,235
Average imputation accuracy for non-genotyped animals (%)	40.55
Minimum imputation accuracy for non-genotyped animals (%)	34.68
Maximum imputation accuracy for non-genotyped animals (%)	53.82

SNP, single nucleotide polymorphism.

**Table 2:** Additive genetic variances and covariances for 305-day milk yield (MY), 305-day fat (Fat), and age at first calving (AFC) estimated using a 3-trait single-step genomic-polygenic model and three sets of SNP genotypes

Variance component	SNP genotypes^1)^					

	SNP Set 1	SE^2)^	SNP Set 2	SE	SNP Set 3	SE
Var (MY) (kg^2^)	179,150.00	23,204.00	170,160.00	22,715.00	155,710.00	22,179.00
Cov (MY, Fat) (kg*%)	−31.10	13.28	−27.34	13.05	−27.56	12.66
Cov (MY, AFC) (kg*mo)	−1.27	98.10	−3.00	96.33	−0.12	93.41
Var (Fat) (%^2^)	0.06	0.01	0.06	0.01	0.06	0.01
Cov (Fat, AFC) (%*mo)	0.14	0.08	0.14	0.08	0.12	0.08
Var (AFC) (mo^2^)	4.90	0.83	4.81	0.81	4.44	0.78

SNP, single nucleotide polymorphism; SE, standard error.

1) SNP Set 1, actual and imputed SNP markers from genotyped animals and no SNP markers from non-genotyped animals; SNP Set 2, actual and imputed SNP markers from genotyped animals plus imputed SNP markers from non-genotyped animals; SNP Set 3, actual and imputed SNP markers from genotyped animals plus actual SNP markers from non-genotyped animals.

2) Repeated sampling approach of Meyer and Houle [24].

**Table 3:** Environmental variances and covariances for 305-d milk yield (MY), 305-d fat (Fat), and age at first calving (AFC) estimated using a 3-trait single-step genomic-polygenic model and three sets of SNP genotypes

Variance component	SNP genotypes^1)^					

	SNP Set 1	SE^2)^	SNP Set 2	SE	SNP Set 3	SE
Var (MY) (kg^2^)	413,410.00	19,936.00	419,950.00	19,805.00	431,860.00	19,577.00
Cov (MY, Fat) (kg*%)	−29.00	12.03	−32.15	11.99	−31.72	11.84
Cov (MY, AFC) (kg*mo)	171.72	85.80	173.18	85.32	169.21	83.80
Var (Fat) (%^2^)	0.18	0.01	0.18	0.01	0.18	0.01
Cov (Fat, AFC) (%*mo)	−0.12	0.07	−0.11	0.07	−0.10	0.07
Var (AFC) (mo^2^)	16.68	0.73	16.72	0.73	17.02	0.71

SNP, single nucleotide polymorphism; SE, standard error.

1) SNP Set 1, actual and imputed SNP markers from genotyped animals and no SNP markers from non-genotyped animals; SNP Set 2, actual and imputed SNP markers from genotyped animals plus imputed SNP markers from non-genotyped animals; SNP Set 3, actual and imputed SNP markers from genotyped animals plus actual SNP markers from non-genotyped animals.

2) Repeated sampling approach of Meyer and Houle [24].

**Table 4:** Phenotypic variances and covariances for 305-day milk yield (MY), 305-day fat (Fat), and age at first calving (AFC) estimated using a 3-trait single-step genomic-polygenic model and three sets of SNP genotypes

Variance component	SNP genotypes^1)^					

	SNP Set 1	SE^2)^	SNP Set 2	SE	SNP Set 3	SE
Var (MY) (kg^2^)	592,560.00	10,975.00	590,110.00	10,828.00	587,570.00	10,723.00
Cov (MY, Fat) (kg*%)	−60.10	6.89	−59.49	6.83	−59.28	6.77
Cov (MY, AFC) (kg*mo)	170.45	46.79	170.19	46.26	169.10	45.81
Var (Fat) (%^2^)	0.24	0.01	0.24	0.01	0.24	0.01
Cov (Fat, AFC) (%*mo)	0.02	0.04	0.02	0.04	0.02	0.04
Var (AFC) (mo^2^)	21.58	0.39	21.53	0.39	21.46	0.39

SNP, single nucleotide polymorphism; SE, standard error.

1) SNP Set 1 = actual and imputed SNP markers from genotyped animals and no SNP markers from non-genotyped animals; SNP Set 2 = actual and imputed SNP markers from genotyped animals plus imputed SNP markers from non-genotyped animals; SNP Set 3 = actual and imputed SNP markers from genotyped animals plus actual SNP markers from non-genotyped animals.

2) Repeated sampling approach of Meyer and Houle [24 ].

**Table 5:** Heritabilities and correlations for 305-day milk yield (MY), 305-day fat (Fat) and age at first calving (AFC) estimated using a 3-trait single-step genomic-polygenic model and three sets of SNP genotypes

Parameters	SNP genotypes^1)^					

	SNP Set 1	SE^2)^	SNP Set 2	SE	SNP Set 3	SE
Heritabilities (MY)	0.30	0.04	0.29	0.04	0.27	0.04
Heritabilities (Fat)	0.26	0.05	0.26	0.05	0.26	0.05
Heritabilities (AFC)	0.23	0.04	0.22	0.04	0.21	0.04
Genetic correlations (MY, Fat)	−0.29	0.12	−0.26	0.13	−0.30	0.14
Genetic correlations (MY, AFC)	0.00	0.11	0.00	0.11	0.00	0.12
Genetic correlations (Fat, AFC)	0.25	0.15	0.25	0.15	0.24	0.16
Environmental correlations (MY, Fat)	−0.11	0.04	−0.12	0.04	−0.11	0.04
Environmental correlations (MY, AFC)	0.07	0.03	0.07	0.03	0.06	0.03
Environmental correlations (Fat, AFC)	−0.07	0.04	−0.07	0.04	−0.06	0.04
Phenotypic correlations (MY, Fat)	−0.16	0.02	−0.16	0.02	−0.16	0.02
Phenotypic correlations (MY, AFC)	0.05	0.01	0.05	0.01	0.05	0.01
Phenotypic correlations (Fat, AFC)	0.01	0.02	0.01	0.02	0.01	0.02

SNP, single nucleotide polymorphism; SE, standard error.

1) SNP Set 1, actual and imputed SNP markers from genotyped animals and no SNP markers from non-genotyped animals; SNP Set 2, actual and imputed SNP markers from genotyped animals plus imputed SNP markers from non-genotyped animals; SNP Set 3, actual and imputed SNP markers from genotyped animals plus actual SNP markers from non-genotyped animals.

2) Repeated sampling approach of Meyer and Houle [24].

**Table 6:** Rank correlations between genomic EBV for 305-day milk yield (MY), 305-day fat (Fat) and age at first calving (AFC) evaluated using a 3-trait single-step genomic-polygenic model and three sets of SNP genotypes

Trait	Rank correlation coefficients±SE^1)^		

	SNP Set 1, SNP Set 2^2)^	SNP Set 1, SNP Set 3	SNP Set 2, SNP Set 3
MY	0.9759±0.0014	0.9769±0.0014	0.9990±0.0003
Fat	0.9470±0.0021	0.9480±0.0021	0.9990±0.0003
AFC	0.9528±0.0020	0.9518±0.0020	0.9990±0.0003

EBV, estimated breeding value; SNP, single nucleotide polymorphism; SE, standard error.

1) All rank correlations were significant (p<0.000^1)^.

2) SNP Set 1, actual and imputed SNP markers from genotyped animals and no SNP markers from non-genotyped animals; SNP Set 2, actual and imputed SNP markers from genotyped animals plus imputed SNP markers from non-genotyped animals; SNP Set 3, actual and imputed SNP markers from genotyped animals plus actual SNP markers from non-genotyped animals.
